# Time-series transcriptomic profiling of larval exsheathment in a model parasitic nematode of veterinary importance

**DOI:** 10.3389/fcell.2023.1257200

**Published:** 2023-11-13

**Authors:** Nikola Palevich, Paul H. Maclean, Richard W. Scott

**Affiliations:** AgResearch Limited, Grasslands Research Centre, Palmerston North, New Zealand

**Keywords:** *Haemonchus contortus*, parasitic nematode, transcriptome, exsheathment, rumen

## Introduction

*Haemonchus contortus* (barber’s pole worm), is one of the most economically important pathogenic nematodes that attacks small ruminants, such as sheep and goats, representing a global animal health issue through drastic losses in livestock. The life cycle of this nematode is comprised of six stages, namely, eggs, first to fourth stage larvae (L1, L2, L3 and L4) and adults (Veglia, 1915). In *Haemonchus contortus* and related nematodes, infective L3s are ingested by the host and enter the rumen where larval exsheathment occurs that marks the transition from the free-living to the parasitic stages of these parasites (Rogers, 1960). Molecular and omics driven studies targeting this key transition phase can help us understand the developmental physiology of this model nematode which can be used to develop both biological and biotechnological control strategies in the future.

There have been earlier transcriptomic and expressed sequence tag (EST) studies describing the differences in transcription between free-living infective (iL3) and parasitic (xL3) third-stage larvae of *H. contortus* (Cantacessi et al., 2010; Laing et al., 2013; Schwarz et al., 2013). However, a major knowledge gap remains amongst the available RNA-seq data sets for this parasitic nematode (Jex et al., 2019). However, all these studies applied the common laboratory practice of the utilization of sodium hypochlorite as the desheathment agent. Recently, a closed *in vitro* parasite culture system that effectively mimics rumen conditions to effectively stimulate exsheathment without chemical interventions has been reported (Bekelaar et al., 2018; Palevich et al., 2022; Palevich et al., 2023a). Briefly, this system involves an increase to rumen temperature (39°C) and a strictly anaerobic environment of predominantly carbon dioxide (CO_2_). The larval exsheathment method used in this study for an RNA-seq approach, has recently been validated as an adaptable technique for multi-omic’ applications of *H. contortus* (Palevich et al., 2022). The aim of our study was to provide the scientific community a detailed time-series transcriptomic description of natural larval exsheathment of *H. contortus*, a valuable resource for future multiomics functional studies investigating larval exsheathment and parasite development.

## Value of the data

• Time-series RNA-seq dataset using an *in vitro* system that effectively reproduces the natural rumen environment fills the remaining gap of the *H. contortus* developmental transcriptome.
• Three distinct expression profiles revealed post-trigger application and identification of the exact time interval that marks the rapid and irreversible population shift in larval exsheathment.
• The presented transcriptomic resource can guide future gene discovery research and the identification of novel compounds with broad-spectrum efficacy that can be used to either trigger premature exsheathment or inhibit the process all together.

## Data

*Haemonchus contortus* is a serious threat in small ruminants causing serious animal production issues worldwide. To improve our understanding of the fundamental genetics of larval exsheathment, the transcriptomes of 100 samples of *H. contortus* L3’s at different stages of the *in vitro* exsheathment process (i.e., 49 time points over 24 h) have been sequenced (RNA-seq) using the Illumina NovaSeq6000 technology. We applied an RNA-seq approach to conduct a time-series transcriptome profiling of infective larvae pre- and post-exsheathment trigger application of the barber’s pole worm. Samples were collected, RNA isolated using TRizol method and stranded mRNA sequencing was carried out using the Illumina NovaSeq6000 platform (Macrogen, Inc.). RNA integrity number (RIN) for all samples were >7 with a total number of 5,918,887,284 clean reads generated. Duplicate runs were performed for all time points (except pre-treatment and *t* = 0 samples that were performed in triplicate) and raw sequences were submitted to Sequence Read Archive (SRA) with data deposited under the GenBank BioProject accession number PRJNA517503. Detailed information regarding BioSample and SRA accession numbers and other related statistics are provided in Supplementary Table S1. Transcripts were mapped against the reference *H. contortus* NZ_Hco_NP v1.0 genome (Palevich et al., 2019b; Palevich et al., 2023b) using STAR v.2.7.1a (Dobin et al., 2013).

**FIGURE 1:**
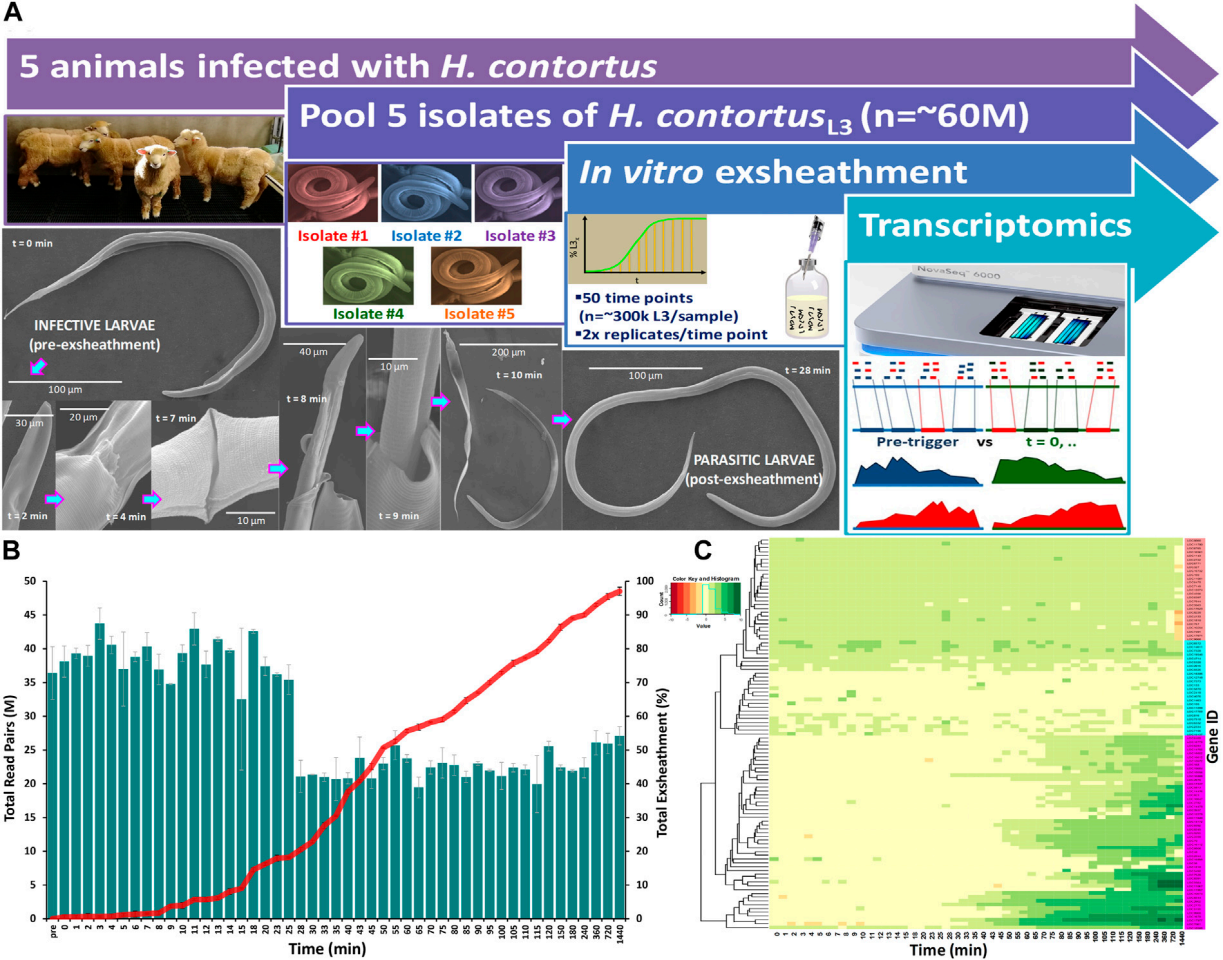
Overview of the study design and experimental procedure. A closed *in vitro* system that effectively reproduces the two basic components of an anaerobic rumen environment (CO_2_ and 39°C) was used to trigger exsheathment (xL3) in *Haemonchus contortus* third-stage infective larvae (iL3) in O_2_-free CO_2_ saturated saline solution **(A)**. Scanning electron micrographs of the exsheathment process with scale bars adjusted according to magnification of each image. Time-series RNA-seq analysis of *Haemonchus contortus* larval exsheathment **(B)**. Comparison of the total numbers of clean sequencing reads *versus* total population exsheathment of L3 larvae. Total reads represent the sum of both unique and duplicate read pairs. Mean of the total exsheathment percentage (±SEM) at each time point across replicates. Top 100 differentially transcribed genes (DTGs) post exsheathment trigger application **(C)**. Standardised relative abundances of inferred gene transcripts (Value) indicating low to high (red to green) relative gene expression (logFC≥2, FDR≥0.05) at each time point, and single linkage clustering. Three distinct profiles of expression were observed within the time-series RNA-Seq data: ‘prolonged/constant’ (pink), ‘oscillating’ (cyan, ≤25 min) and ‘ramped’ (purple, ≥28 min) expression for the majority of time intervals.

The transcriptome data has revealed significant differences in the overall gene expression profiles of the parasite populations at different stages of the exsheathment process. A comparison of the total numbers of sequencing read pairs at each time point reveals a dramatic decrease at the 28-min interval (Figure 1B). This transition indicates a strong negative correlation between the total numbers of exsheathed *H. contortus* L3 larvae (xL3) and sequencing read pairs observed with respect to the total population of exsheathed xL3’s over time for these samples, that was strongest at the 28-min interval indicating the rapid and irreversible population shift in exsheathment.

**FIGURE 2:**
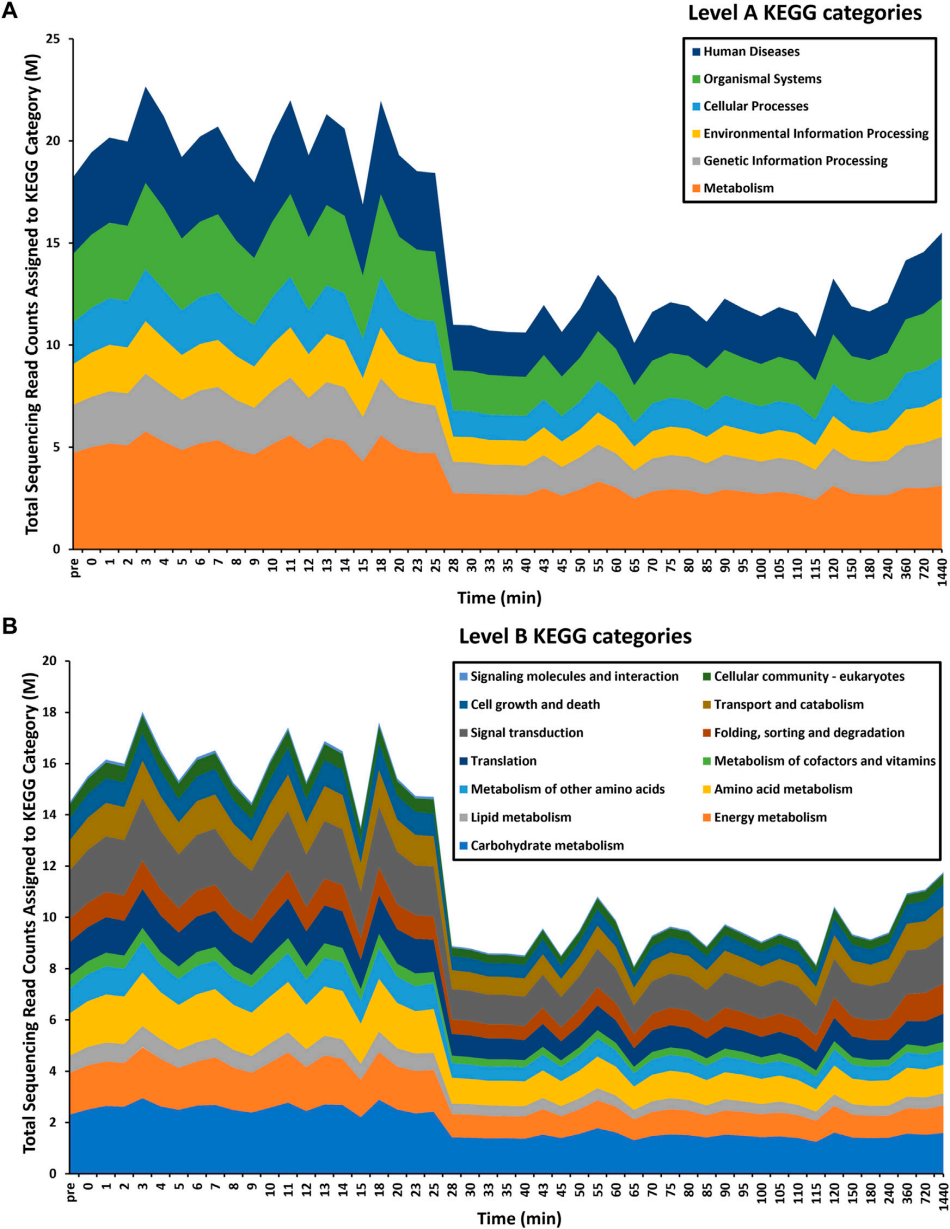
Time-series functional profiling of larval exsheathment in *Haemonchus contortus*. For each transcriptomic dataset, only the top level-A **(A)** and corresponding level-B **(B)** KEGG pathways are depicted. The total sequencing reads assigned to KEGG categories are averages across each time point.

Overall, a total of 863 significantly (logFC≥2, FDR≥0.05) differentially transcribed genes (DTGs) post trigger application with three distinct patterns or profiles of expression observed (Figure 1C). A large proportion of these DTGs are predicted to encode hypothetical proteins or proteins that are yet to be characterized (Palevich et al., 2018), and genes that were only upregulated at the final 720 and 1,440 min (12 and 24 h) time intervals. Further, a significant decrease in the total sequencing read counts assigned to Kyoto Encyclopedia of Genes and Genomes (KEGG) pathways was also observed at the 28-min interval (Figure 2). Across all time points, the categories ‘signal transduction’ and ‘carbohydrate metabolism’ were the two most abundant B-level KEGG functions (Figure 2B). The presented time-series RNA-seq dataset of natural larval exsheathment in *H. contortus* can be used for applications such as gene discovery or comparison with genomes and/or transcriptomes of other parasitic nematodes of veterinary importance to understand the molecular mechanisms driving their parasitism.

## Materials and methods

### Nematode production and procurement

Pure cultures of *H. contortus* third-stage larvae (L3) were maintained by regular passage through five otherwise parasite-free lambs housed indoors at the AgResearch’s Grasslands campus (Figure 1A). L3 were cultured in fresh faecal material containing eggs collected into faecal bags on infected sheep. Faeces were pooled and mixed with vermiculite then placed in trays, moistened with tap water (at 20°C), covered and cultured for 10 days at 22°C–24°C. A modified Baermann technique was used to clean and separate the larvae from faeces (Viglierchio and Schmitt, 1983). Briefly, approximately 150 g of faeces was enclosed in paper facial tissues and suspended over a large conical measuring flask filled with unchlorinated water. The samples were incubated for 20 h and larvae washed by their movement through the apparatus. The faeces were then removed and the liquid carefully siphoned to a remaining volume of about 20 mL. The resulting L3s and volume were examined in a counting chamber (Whitlock S.F.E.L.O., Australia; volume 2 mL) at ×100 magnification and stored in tap water at 10°C until required. A single batch of larvae were used for all experiments to account for any batch-related confounding factors with larval viability and motility checked microscopically prior to any further experimentation.

The provenance of genomic DNA was verified with a 100% similarity identity to the representative chromosome-level genome of the anthelmintic-susceptible *H. contortus* field strain (Palevich et al., 2019a; Palevich et al., 2019b; Palevich et al., 2020), by automated Sanger sequencing of the second internal transcribed spacer (ITS-2) of nuclear ribosomal DNA following PCR amplification from genomic DNA.

### *In vitro* larval exsheathment, sample collection and electron microscopy

Prior to *in vitro* testing, pooled L3 cultures were cleaned using autoclaved phosphate buffered saline (1× PBS) solution (137 mM NaCl, 2.7 mM KCl, 8 mM Na_2_HPO_4_, and 2 mM KH_2_PO_4_, pH 7.4) and acclimated overnight to room temperature by gravity migration filtration through nylon mesh (pore size 20 μm). Larval viability and motility were checked microscopically and quantified using a Petroff-Hausser chamber (Hausser Scientific) according to the manufacturer’s instructions.

In this study, we triggered larval exsheathment using a modification of the method described by Bekelaar et al., 2018, in a ‘closed’ *in vitro* system that effectively and reproducibly simulates the physiological conditions of the rumen (39°C and high CO_2_ concentration) as previously described (Palevich et al., 2022; Palevich et al., 2023a). Briefly, anaerobic 1× PBS solution was mixed in boiling dH_2_O and cooled to room temperature under a continuous flow of O_2_-free CO_2_. Once cooled, the PBS solution was transferred to 100 mL serum bottles in 70 mL aliquots and flushed with CO_2_ for 1 h. The bottles were sealed with butyl rubber bungs and aluminium crimp caps before being autoclaved at 121°C for 20 min. Larval cultures were transferred anaerobically (approximately 300,000 L3/sample) using a O_2_-free CO_2_-flushed 3 mL syringe and wide bore hypodermic needle (16 G thickness and length of 1–1/2 inch, BD), into aluminium-wrapped and pre-warmed serum bottles containing 70 mL of autoclaved PBS and incubated anaerobically at 39°C with gentle horizontal shaking at 75 rpm for up to 24 h in darkness.

Exsheathment of L3 larvae (xL3) was determined retrospectively by either complete or partial loss of the sheath and was measured by additional sub-sampling of each replicate beginning at *t* = 0 min up to 24 h after incubation at 39°C and CO_2_ anaerobic conditions. At each time point, contents were thoroughly mixed before 1 mL was transferred anaerobically to a 24-well plate and exsheathment enumerated. The numbers of xL3s in each subsample was quantified via 300-fold dilution of sample to another 24-well plate containing dH_2_O to yield approximately 300 larvae per replicate. Larvae were killed by the addition of 1 drop of 3% helminthological iodine solution (Lugol’s solution) and exsheathment enumerated. Samples were collected anaerobically from each time point, snap-frozen in liquid nitrogen and stored at −80°C until further processing.

Scanning electron microscopy (SEM) was performed as previously described (Palevich et al., 2019b; Palevich et al., 2022). Briefly, cryopreserved worms were gently spun, washed 3x in PBS and fixed in SEM primary fixative (3% glutaraldehyde, 2% formaldehyde in 0.1 M Phosphate Buffer pH 7.2) for 2 days at room temperature. Samples were dehydrated in a graded ethanol series, i.e. 25%, 50%, 75%, and 95% for 10–15 min each and 2× in 100% ethanol for 1 h, then Critical Point (CP) dried using liquid CO_2_ and mounted onto an aluminium specimen support stub using double-sided adhesive tape. Samples were sputter coated with gold (200 s) and observed using a FEI Quanta 200 Environmental Scanning electron microscope with energy dispersive x-ray spectroscopy (EDAX) module.

### Total RNA extraction, library preparation and RNA-Seq

Snap-frozen samples containing 100 µL of packed worms were lysed using an 18 V drill loaded with a disposable RNAase-free polypropylene Micro-Pestle (Qiagen) until the mix is ground to a fine white powder. To the ground sample 250 µL of pre-warmed (40°C) TRizol (Life Technologies) was added and mixed thoroughly according to the manufacturer’s instructions, snap-frozen in liquid N_2_ and the homogenization of snap frozen samples in TRizol was repeated for five rounds in total to ensure complete disruption of the sample. To the homogenized sample, 750 µL of pre-warmed TRizol and 0.1 volume of chloroform were added, thoroughly mixed and centrifuged at 20,000 × *g* for 10 min at 4°C. The upper aqueous phase was transferred into a new Eppendorf tube and an equal volume of isopropanol and 0.1 volume of 3 M sodium acetate (pH 5.5) were added and gently mixed, and the mixture was stored at −20°C overnight. The RNA pellets were precipitated with ethanol, re-suspended in nuclease-free water (Life Technologies) and DNase I treated. RNA yield and quality were assessed using the Bioanalyzer 2,100 with the RNA 6000 Nano assay reagent kit from Agilent (Santa Clara, CA) and stored at −80°C. NGS sequencing libraries were generated from 1 μg of total RNA using TruSeq Stranded RNA Sample Prep Kit (Illumina) according to the manufacturer’s protocol. The resulting cDNA libraries were then paired-end sequenced (2 × 100 bp) using the NovaSeq6000 instrument (Macrogen, Inc.), to produce 2,959,443,642 read pairs in total with an average of 29,594,436 per sample.

### Functional annotation and differential expression analysis of time-series RNA-seq data

Complete paired-end sequences were obtained and converted into FASTQ files (forward and reverse) using bcl2fastq (Illumina) package from Macrogen. Adaptor sequences, minimum length (36 bp), bad fraction (>10%) and low-quality bases with PHRED scores (Q) ≤ 20, were removed and paired-end reads cleaned using Trimmomatic v0.4 (Bolger et al., 2014). The reads were mapped against the reference of *H. contortus* NZ_Hco_NP v1.0 genome (Palevich et al., 2019b; Palevich et al., 2023a) using STAR version v2.7.1a (Dobin et al., 2013). The counts were compiled and tabulated, and a differential expression was performed using the DESeq2 v1.30.2 package (Love et al., 2014) with default parameters. A differentially transcribed gene (DTG) was defined as a transcript with an absolute log fold change ≥2 and an FDR of less than 0.05. The “top” DGTs were chosen by their ranking in absolute fold change and displayed a consistent level expression for the majority of time intervals. The sample features, GenBank BioSample and Sequence Read Archive (SRA) accession numbers for the time-series exsheathment transcriptomes of *H. contortus* are listed in Supplementary Table S1.

Possible protein coding regions within the genome-based transcripts were identified using the TransDecoder program v5.5.0 implemented in TRINITY v2.14.0 (Grabherr et al., 2011). The protein-coding regions were searched against the NCBI NR protein sequence database using the *blastp* function of DIAMOND v2.0.6 (Buchfink et al., 2015) with the output format of XML being specified. The results were imported into OmicsBox v1.4.11 (https://www.biobam.com), where the Blast2GO (Conesa et al., 2005) annotation functions were used with default settings. InterProScan v5.50–84.0 (Jones et al., 2014) and EggNOG-Mapper v1.0.3 with EggNOG v5.0.0 (Huerta-Cepas et al., 2018) were further used with default settings to annotate the predicted proteins. For analysis of KEGG biological pathways, gene abundance tables were generated by alignment of the sequencing reads to the NCBI NR database using the *blastx* function of DIAMOND v2.0.6 (Buchfink et al., 2015) aligner. The results were “MEGANised” using the tools provided with MEGAN6 Ultimate Edition (Huson et al., 2016) and loaded into the MEGAN software to assign putative functions to the DIAMOND alignment files for level A and B KEGG categories (Supplementary Table S2).

## Data Availability

The datasets presented in this study can be found in online repositories. The names of the repository/repositories and accession number(s) can be found in the article/Supplementary Material.
